# A Fast Space-Time Adaptive Processing Algorithm Based on Sparse Bayesian Learning for Airborne Radar

**DOI:** 10.3390/s22072664

**Published:** 2022-03-30

**Authors:** Cheng Liu, Tong Wang, Shuguang Zhang, Bing Ren

**Affiliations:** National Lab of Radar Signal Processing, Xidian University, Xi’an 710071, China; m18392121020@163.com (C.L.); SG_Zhang12138@163.com (S.Z.); renb0327@163.com (B.R.)

**Keywords:** space-time adaptive processing, airborne radar, sparse recovery/representation, computational complexities, sparse Bayesian learning, group sparse

## Abstract

Space-time adaptive processing (STAP) plays an essential role in clutter suppression and moving target detection in airborne radar systems. The main difficulty is that independent and identically distributed (i.i.d) training samples may not be sufficient to guarantee the performance in the heterogeneous clutter environment. Currently, most sparse recovery/representation (SR) techniques to reduce the requirement of training samples still suffer from high computational complexities. To remedy this problem, a fast group sparse Bayesian learning approach is proposed. Instead of employing all the dictionary atoms, the proposed algorithm identifies the support space of the data and then employs the support space in the sparse Bayesian learning (SBL) algorithm. Moreover, to extend the modified hierarchical model, which can only apply to real-valued signals, the real and imaginary components of the complex-valued signals are treated as two independent real-valued variables. The efficiency of the proposed algorithm is demonstrated both with the simulated and measured data.

## 1. Introduction

Space-time adaptive processing (STAP) has the capability to detect slow-moving targets that might otherwise be swallowed up by the strong sidelobe clutter. The performance of STAP filter is dependent on the accuracy of the clutter plus noise covariance matrix (CNCM) of the cell under test (CUT) [1]. According to the well-known Reed-Mallet-Brennan (RMB) rule [2], to achieve the steady performance, the number of the independent and identically distributed (i.i.d) secondary range cells used to estimate CNCM is no less than twice the system degrees of freedom (DOF). However, it is hard to obtain enough i.i.d samples in practice because of array geometry structures, nonhomogeneous clutter environment and so on [1]. Knowing how to improve the performance of STAP with limited samples has been a hot topic until now.

Reduced-dimension (RD) [3] and reduced-rank (RR) [4] algorithms are proposed to improve the performance of STAP with limited secondary samples. Even though they are easy to implement, their performance gets worse when the number of secondary samples is smaller than twice the rank of clutter [5].

Apart from RD and RR algorithms, some other algorithms [6,7,8,9,10] are proposed and succeed in suppressing clutter in theory. However, they face some disadvantages in practice. Sufficiently, (1) direct data domain (DDD) algorithms in [6] that achieve enough samples from the CUT suffer from aperture loss; and (2) knowledge-aided (KA) algorithms in [7,8,9,10] state that the accurate prior knowledge must be required in advance. Since the cost to achieve the accurate prior knowledge is expensive and the prior knowledge changes with time, KA algorithms are not widely used in practical applications.

Over the past twenty years, sparse recovery/representation (SR) algorithms have received continuous attention in STAP [11,12,13,14,15] because they have enormous potential to estimate the clutter spectrum with limited samples. The sparse Bayesian learning (SBL)-type algorithms are robust and have drawn continuous attention in the last five years.

The SBL algorithm was proposed by Tipping in [16]. Wipf enhanced it in the single measurement vector (SMV) case in [17] and then the multiple measurement vectors (MMV) case in [18]. Due to the excellent performance of SBL algorithms, the fast marginal likelihood maximization (FMLM) algorithm [19], the Bayesian compressive sensing (BCS) algorithm [20], the multitask BCS (M-BCS) algorithm [21], and other Bayesian algorithms [22,23,24] were proposed by researchers in the following years. SBL was firstly introduced into STAP with MMV, defined as the M-SBL-STAP algorithm in [25], by Duan to estimate CNCM and Wang improved the fast convergence of the algorithm in [26]. SBL has also been used to solve some common problems in STAP in the last five years, such as off-grid in [27] and discrete interference in [28]. However, the Bayesian algorithms aforementioned have one or more of the following disadvantages: (a) high computational cost, (b) inaccurate estimation of the noise variance and (c) inapplicability to complex-valued signals.

In this paper, to improve the computational efficiency of the M-SBL-STAP algorithm, we extend the FMLM algorithm to the conventional M-SBL-STAP algorithm. The real and imaginary components of the complex-valued signals are treated as two independent real-valued variables in order to extend the modified hierarchical model. Simulation results with both simulated and Mountain-Top data demonstrate that the proposed algorithm can achieve high computational efficiency and good performance.

The main contributions of this paper can be listed as follows:

1. We extend the FMLM algorithm into M-SBL-STAP for the purpose of identifying the support space of the data, i.e., the atoms whose corresponding hyper-parameters are non-zero. After support space identification, the dimensions of the effective problems are drastically reduced due to sparsity, which can reduce computational complexities and alleviate memory requirements.
2. According to [18], we have no access to obtain the accurate value of the noise variance. Instead of estimating the noise variance, we extend the modified hierarchical model, introduced in Section 4, to the SBL framework.
3. Although the hierarchical models apply to the real-valued signals, they cannot be extended directly to the complex-valued signal according to [29,30]. The data needed to be dealt with in STAP are all complex-valued. To solve the problem, we transform sparse complex-valued signals into group sparse real-valued signals.

Notation: In this article, scalar quantities are denoted with italic typeface. Boldface small letters are reserved for vectors, and boldface capital letters are reserved matrices. The i-th entry of a vector x is denoted by $x_{i}$, while $A_{i}$ and $A_{ij}$ denote the i-th row and $\left(i,j\right)-\mathrm{th}$ element of a matrix A, respectively. The symbols ${\left(•\right)}^{T}$ and ${\left(•\right)}^{H}$ denote the matrix transpose and conjugate transpose, respectively. The symbols ${\left\|•\right\|}_{1}$, ${\left\|•\right\|}_{2}$ and ${\left\|•\right\|}_{F}$ are reserved for $\ell_{1}$, $\ell_{2}$ and Frobenius ($\ell_{F}$) norms, respectively. ${\left\|•\right\|}_{0}$ is reserved for $\ell_{0}$ pseudo-norm. ${\left\|•\right\|}_{2,0}$ stands for a mixed norm defined as the number of non-zero elements of the vector formed by the $\ell_{2}$ norm of each row vector. The symbol $\left|•\right|$ is reserved for the determinant. The notations I, 0 and 1 represent the identity matrix, the all zero matrix and the all one matrix, respectively. The expectation of a random variable is denoted as $Ε\left(•\right)$.

## 2. Background and Problem Formulation

### 2.1. STAP Signal Model for Airborne Radar

Consider an airborne pulsed-Doppler radar system with a side-looking uniform linear array (ULA) consisted of N elements. A coherent burst of M pulses is transmitted at a constant pulse repetition frequency (PRF) in a coherent processing interval (CPI). The complex sample at the CUT from the n-th element and the m-th pulse is denoted as $x_{mn}$, and the data for the CUT can be written as a MN×1 vector x, termed a space-time snapshot.

(1) $$x={\left[x_{11},x_{12},\cdots ,x_{1N},x_{21},\cdots ,x_{MN}\right]}^{T}$$

Radar systems need to ascertain whether the targets are present in the CUT; thus, target detection is formulated into a binary hypothesis problem: the hypothesis $H_{0}$ represents target absence and the other hypothesis $H_{1}$ represents target presence.

(2) $$\left\{\begin{array}{cc} x=x_{c}+n & H_{0}\\ x=\alpha_{t}S_{t}+x_{c}+n & H_{1} \end{array}\right.$$

where $\alpha_{t}$ is the target amplitude and $S_{t}\in {\mathbb{C}}^{MN\times 1}$ is the space-time vector of the target. The vector $n\in {\mathbb{C}}^{MN\times 1}$ is the thermal noise, which is uncorrelated both spatially and temporally. A general model for the space-time clutter $x_{c}\in {\mathbb{C}}^{MN\times 1}$ is

(3) $$x_{c}=\sum_{k=1}^{N_{c}}\alpha_{k}\upsilon \left(f_{d,k},f_{s,k}\right)$$

(4) $$\upsilon_{\mathrm{st}}\left(f_{d,k},f_{s,k}\right)=\upsilon_{t}\left(f_{d,k}\right)⊗\upsilon_{s}\left(f_{s,k}\right)$$

(5) $$\upsilon_{t}\left(f_{d,k}\right)={\left[1,\exp\left(j\pi f_{d,k}\right),\ldots ,\exp\left(j\pi \left(M-1\right)f_{d,k}\right)\right]}^{T}$$

(6) $$\upsilon_{s}\left(f_{s,k}\right)={\left[1,\exp\left(j\pi f_{s,k}\right),\ldots ,\exp\left(j\pi \left(N-1\right)f_{s,k}\right)\right]}^{T}$$

where $\alpha_{k}$ denotes the random amplitude of the echo from the corresponding clutter patch; $N_{c}$ denotes the number of clutter patches in a clutter ring; $\upsilon_{\mathrm{st}}$, $\upsilon_{t}$ and $\upsilon_{s}$ denote space-time steering vector, temporal steering vector and spatial steering vector, respectively; and $f_{d,k}$ and $f_{s,k}$ denote the corresponding normalize temporal frequency and spatial frequency, respectively.

According to the linearly constrained minimum variance (LCMV) criterion, the optimal STAP weight vector is

(7) $$w_{\mathrm{opt}}=\frac{R_{c+n}^{-1}S_{t}}{S_{t}^{H}R_{c+n}^{-1}S_{t}}$$

where the CNCM $R_{c+n}$ is expressed as

(8) $$R_{c+n}=R_{c}+R_{n}=E\left\{x_{c}x_{c}^{H}\right\}+\sigma^{2}I$$

### 2.2. SR-STAP Model and Principle

Discretize the space-time plane uniformly into $K=N_{S}N_{d}$ grids, where $N_{S}=\varphi_{S}N$ $\left(\varphi_{S}>1\right)$ denotes the number of normalized spatial frequency bins and $N_{d}=\varphi_{d}M$ $\left(\varphi_{d}>1\right)$ denotes the number of normalized Doppler frequency bins. Each grid corresponds to a space-time steering vector $\upsilon_{k}\;,\;\left(k=1,2,\cdots ,\;K\right)$. The dictionary $D\in {\mathbb{C}}^{MN\times K}$ used in STAP is the collection of space-time steering vectors of all grids.

(9) $$D=\left[\upsilon_{1},\upsilon_{2},\cdots \;,\upsilon_{K}\right]$$

The signal model in STAP can be cast in the following form

(10) X=DA+n

where $X=\left[x_{1},x_{2},\cdots \;,x_{L}\right]$; $A\in {\mathbb{C}}^{K\times L}$ denotes sparse coefficient matrix; non-zero elements indicate the presence of clutter on the space-time profile; L denotes the number of the range gates in the data; and $n\in {\mathbb{C}}^{MN\times L}$ denotes zero-mean Gaussian noise.

In sparse signal recovery algorithms with MMV, our goal is to represent the measurement X, which is contaminated by noise as a linear combination of as few dictionary atoms as possible. Therefore, the objective function is expressed as

(11) $$A^{*}=\underset{A}{\arg\min}{\left\|A\right\|}_{2,0},\;s.t.\;{\left\|X-DA\right\|}_{F}^{2}\leq \varepsilon$$

where ε is the noise error allowance.

## 3. M-SBL-STAP Algorithm

### 3.1. Sparse Bayesian Learning Formulation

In the SBL framework, ${\left\{x_{l}\right\}}_{l=1}^{L}$ are assumed as i.i.d training snapshots. The noise is submitted to white complex Gaussian distribution with unknown power $\sigma^{2}$. The likelihood function of the measurement vectors can be denoted as

(12) $$p\left(X|A,\sigma^{2}\right)={\left(\pi \sigma^{2}\right)}^{-MNL}\exp\left[-\sigma^{-2}\sum_{l=1}^{L}{\left\|x_{l}-Da_{l}\right\|}^{2}\right]$$

Since the training snapshots are i.i.d, each column in A is independent and shares the same covariance matrix. Assign to each column in A with a zero-mean Gaussian prior

(13) $$a_{l}\sim CN\left(0,\Gamma \right),l=1,2,\cdots ,L$$

where $0\in {\mathbb{C}}^{K\times 1}$ is a zero vector and $\Gamma =\mathrm{diag}\left(\gamma \right)$. $\gamma =\left\{\gamma_{1},\gamma_{2},\cdots ,\gamma_{K}\right\}$ are unknown variance parameters corresponding to sparse coefficient matrix. The prior of A can be expressed as

(14) $$p\left(A|\Gamma \right)=\pi^{-KL}\Gamma^{-L}\exp⟮-\sum_{l=1}^{L}a_{l}^{H}\Gamma^{-1}a_{l}⟯$$

Combining likelihood with prior, the posterior density of A can be easily expressed as

(15) $$p\left(A|X,\Gamma ,\sigma^{2}\right)=\frac{p\left(X|A,\sigma^{2}\right)p\left(A|\Gamma \right)}{\int p\left(X|A,\sigma^{2}\right)p\left(A|\Gamma \right)dA}$$

modulated by the hyper-parameters γ and $\sigma^{2}$. To find the hyper-parameters γ and $\sigma^{2}$, which are enough accurate to estimate the CNCM, the most common method is the expectation maximization (EM) algorithm. The EM algorithm is divided into two parts: E-step and M-step. t represents the sequence number of the current iteration. At the E-step: treat A as hidden data, and the posterior density can be described with hyper-parameters γ and $\sigma^{2}$.

(16) $$\begin{array}{l} p\left(A^{\left(t+1\right)}|X,\Gamma^{\left(t\right)},{\left\{\sigma^{2}\right\}}^{\left(t\right)}\right)=\frac{p\left(X,A^{\left(t+1\right)}|\Gamma^{\left(t\right)},{\left\{\sigma^{2}\right\}}^{\left(t\right)}\right)}{p\left(X|\Gamma^{\left(t\right)},{\left\{\sigma^{2}\right\}}^{\left(t\right)}\right)}\\ =\pi^{-KL}{\left|\Sigma^{\left(t+1\right)}\right|}^{-L}\exp\left[-\sum_{l=1}^{L}{\left(a_{l}^{\left(t+1\right)}-\mu_{l}^{\left(t+1\right)}\right)}^{H}{\left(\Sigma^{\left(t+1\right)}\right)}^{-1}\left(a_{l}^{\left(t+1\right)}-\mu_{l}^{\left(t+1\right)}\right)\right] \end{array}$$

with covariance and mean given by

(17) $$\Sigma^{\left(t+1\right)}={\left({\left\{\sigma^{-2}\right\}}^{\left(t\right)}D^{H}D+\Lambda^{\left(t\right)}\right)}^{-1}$$

(18) $$\mu^{\left(t+1\right)}={\left\{\sigma^{-2}\right\}}^{\left(t\right)}\Sigma^{\left(t+1\right)}D^{H}X$$

where

(19) $$\Lambda^{\left(t\right)}={\left\{\Gamma^{\left(t\right)}\right\}}^{-1}=\mathrm{diag}⟮\left[\frac{1}{\gamma_{1}^{\left(t\right)}},\frac{1}{\gamma_{2}^{\left(t\right)}},\cdots ,\frac{1}{\gamma_{K}^{\left(t\right)}}\right]⟯$$

(20) $$\mu^{\left(t+1\right)}=\left[\mu_{1}^{\left(t+1\right)},\mu_{2}^{\left(t+1\right)},\cdots ,\mu_{L}^{\left(t+1\right)}\right]$$

At the M-step, we update the hyper-parameters by maximizing the expectation of $p\left(X,A^{\left(t+1\right)}|\Gamma^{\left(t\right)},{\left\{\sigma^{2}\right\}}^{\left(t\right)}\right)$.

(21) $$\left[\gamma^{\left(t+1\right)},{\left\{\sigma^{2}\right\}}^{\left(t+1\right)}\right]=\underset{\Gamma^{\left(t\right)},{\left\{\sigma^{2}\right\}}^{\left(t\right)}}{\mathrm{argmax}}E\left[\ln p\left(X,A^{\left(t+1\right)}|\Gamma^{\left(t\right)},{\left\{\sigma^{2}\right\}}^{\left(t\right)}\right)\right]$$

where

(22) $$\gamma_{i}^{\left(t+1\right)}=\frac{1}{L}\sum_{l=1}^{L}{\left\|\mu_{l}^{\left(t+1\right)}\left(i\right)\right\|}_{2}^{2}+\Sigma_{ii}^{\left(t+1\right)}$$

(23) $${\left\{\sigma^{2}\right\}}^{\left(t+1\right)}=\frac{\frac{1}{L}\sum_{l=1}^{L}{\left\|x_{l}-D\mu_{l}^{\left(t+1\right)}\right\|}_{2}^{2}}{NM-K+\sum_{k=1}^{K}\frac{\Sigma_{kk}^{\left(t+1\right)}}{\gamma_{k}^{\left(t\right)}}}$$

The M-SBL-STAP algorithm is shown in Algorithm 1.
**Algorithm 1:** Pseudocode for the M-SBL-STAP algorithm.Step 1: Input: the clutter data X, the dictionary DStep 2: Initialization: initial the values of γ and $\sigma^{2}$.Step 3: E-step: update the posterior moments $\Sigma^{\left(t+1\right)}$ and $\mu^{\left(t+1\right)}$ using (17) and (18).Step 4: M-step: update $\gamma^{\left(t+1\right)}$ and ${\left\{\sigma^{2}\right\}}^{\left(t+1\right)}$ using (22) and (23).Step 5: Repeat step 3 and step 4 until a stopping criterion is satisfied.Step 6: Estimate the CNCM R by the formula       $R=\frac{1}{L}\sum_{l=1}^{L}\sum_{k=1}^{K}\left({\left\|\mu_{l,k}^{*}\right\|}^{2}\right)\upsilon_{k}\upsilon_{k}^{H}+\beta Ι$
  where β is a load factor and the symbol * represents the stopping criterion.Step 7: Compute the space-time adaptive weight w using (7).Step 8: The output of the M-SBL-STAP algorithm is $Z=w^{H}X$.

### 3.2. Problem Statement of the M-SBL-STAP Algorithm

At the E-step in each iteration, the inversion of a K×K matrix, which brings high computational complexities in the order of $o\left(K^{3}\right)$, is required to be calculated when update covariance Σ (17). K is the number of the atoms in the dictionary, and the value of K is usually large. If we avoid calculating the matrix inversion of a K×K matrix, the computational complexities can be reduced a lot.

At the M-step in each iteration, the noise variance $\sigma^{2}$ needs to be estimated. However, it has been demonstrated in [18] that $\sigma^{2}$ estimated by (23) can be extremely inaccurate when the dictionary is structured and K≥NM. We can see in [21] that $\sigma^{2}$ is a nuisance parameter in the iterative procedure and an inappropriate value may contaminate the convergence performance of the algorithm.

We extend a modified hierarchical model to the SBL framework, which aims at integrating $\sigma^{2}$ out instead of estimating $\sigma^{2}$. However, the modified model, which applies to the real-valued signals, cannot be directly extended to complex-valued signals.

In the next sections, we introduce the proposed algorithm to solve the above problems. The proposed algorithm is defined as M-FMLM-STAP algorithm.

## 4. The Proposed M-FMLM-STAP Algorithm

### 4.1. Modified Hierarchical Model

In [16], the scholars define the following hierarchical hyperpriors over Γ, as well as the noise variance $\sigma^{2}$.

(24) $$p\left(\Gamma |c,d\right)=\prod_{k=1}^{K}\mathrm{Gamma}⟮\frac{1}{\gamma_{k}}|c,d⟯$$

(25) $$p\left(\sigma^{2}|a,b\right)=\mathrm{Gamma}\left(\sigma^{-2}|a,b\right)$$

where $\mathrm{Gamma}\left(\alpha |a,b\right)=\Gamma {\left(a\right)}^{-1}b^{a}\alpha^{a-1}e^{-b\alpha }$ and the ‘gamma function’ $\Gamma \left(a\right)=\int_{0}^{\infty }t^{a-1}e^{-t}dt$. It has been demonstrated in [16] that an appropriate choice of the shape parameter a and the scale parameter b encourages the sparsity of the coefficient matrix.

In the STAP framework, CNCM can be expressed as

(26) $$R_{c+n}=D\Gamma D+\sigma^{2}I$$

We can translate STAP weight vector in the form

(27) $$\begin{array}{ll} w_{\mathrm{opt}} & =\mu R_{c+n}^{-1}S_{t}\\ & =\lambda {⟮D\frac{\Gamma }{\sigma^{2}}D+I⟯}^{-1}S_{t}\\ & =\lambda {\tilde{R}}^{-1}S_{t} \end{array}$$

where λ and μ are constants.

The above analysis shows that $\tilde{R}$ is equivalent to $R_{c+n}$ in the performance of clutter suppression. Thus, including $\sigma^{2}$ in the prior of Γ, each component of A is defined as a zero-mean Gaussian distribution and the modified hierarchical model follows (28) and (29).

(28) $$p\left(A|\Gamma^{\prime },\sigma^{2}\right)=\prod_{l=1}^{L}\prod_{k=1}^{K}CN\left(a_{l,k}|0,\gamma_{k}\right)=\prod_{l=1}^{L}\prod_{k=1}^{K}CN\left(a_{l,k}|0,\sigma^{2}\gamma_{k}^{\prime }\right)$$

(29) $$p\left(\sigma^{2}|a,b\right)=\mathrm{Gamma}\left(\sigma^{-2}|a,b\right)$$

where $\Gamma^{\prime }=\mathrm{diag}\left(\gamma^{\prime }\right)=\mathrm{diag}\left(\gamma_{1}^{\prime },\gamma_{2}^{\prime },\cdots ,\gamma_{2K}^{\prime }\right)=\frac{\Gamma }{\sigma^{2}}$.

### 4.2. Application of the Modified Hierarchical Model to Complex-Valued Signals

However, although both of the original and the modified models apply to real-valued signals, they cannot be directly extended to complex-valued signals. To solve the above problem, the real and imaginary components of the complex-valued signals are treated as two independent real-valued variables.

(30) yl=RexlImxl

The new signal model is expressed as

(31) Y=ΨB+N

where $Y=\left[y_{1},y_{2},\cdots \;,y_{L}\right]\in {\mathbb{R}}^{2MN\times L}$ is the new data model, $B=\left[b_{1},b_{2},\cdots \;,b_{L}\right]\in {\mathbb{R}}^{2K\times L}$ is the new sparse coefficient matrix and $N\in {\mathbb{R}}^{2MN\times L}$ is the new noise matrix in new model. The new dictionary $\Psi \in {\mathbb{R}}^{2MN\times 2K}$ is expressed as

(32) Ψ=Reυ1−Imυ1⋯ReυK−ImυKImυ1Reυ1⋯ImυKReυK

while the new covariance, mean and hyper-parameter in the modified model are expressed as

(33) $$\Sigma ={\left(\sigma^{-2}\Psi^{T}\Psi +\Gamma^{-1}\right)}^{-1}$$

(34) $$\mu =\sigma^{-2}\Sigma \Psi^{T}y$$

(35) $$\Gamma =\mathrm{diag}\left(\gamma_{1},\gamma_{2},\cdots ,\gamma_{K},\gamma_{K+1},\gamma_{K+2},\cdots ,\gamma_{2K}\right)$$

where $\gamma_{2i-1}=\gamma_{2i},\;i=1,2,\cdots ,K$. The signal $y_{l}$ is group sparse because the entries in each group, saying $\gamma_{\left\{i\right\}}=\left\{\gamma_{2i-1},\gamma_{2i}\right\},\;i=1,2,\cdots ,K$, are either zero or nonzero simultaneously.

In order to avoid calculating $\sigma^{2}$ in (33) and (34), we extend the modified hierarchical model to the SBL framework. The new real-valued likelihood function can be expressed as

(36) $$\begin{array}{ll} p\left(Y|B,\sigma^{2}\right) & ={\left(2\pi \sigma^{2}\right)}^{-\frac{2MNL}{2}}\exp\left[-\frac{1}{2\sigma^{2}}\sum_{l=1}^{L}{\left\|y_{l}-\Psi b_{l}\right\|}^{2}\right]\\ & ={\left(2\pi \sigma^{2}\right)}^{-MNL}\exp\left[-\frac{1}{2\sigma^{2}}\sum_{l=1}^{L}{\left\|y_{l}-\Psi b_{l}\right\|}^{2}\right] \end{array}$$

and the new prior of B can be expressed as

(37) $$\begin{array}{ll} p\left(B|\Gamma^{\prime },\sigma^{2}\right) & ={\left(2\pi \right)}^{-\frac{2KL}{2}}\Gamma^{-\frac{L}{2}}\exp⟮-\frac{1}{2}\sum_{l=1}^{L}b_{l}^{T}\Gamma^{-1}b_{l}⟯\\ & ={\left(2\pi \right)}^{-KL}{\left(\sigma^{2}\Gamma^{\prime }\right)}^{-\frac{L}{2}}\exp⟮-\frac{1}{2}\sum_{l=1}^{L}b_{l}^{T}{\left(\sigma^{2}\Gamma^{\prime }\right)}^{-1}b_{l}⟯ \end{array}$$

Combining the new likelihood and prior, the new posterior density of B can be expressed as

(38) $$p\left(B|Y,\Gamma^{\prime },\sigma^{2}\right)=\frac{p\left(Y|B,\sigma^{2}\right)p\left(B|\Gamma^{\prime },\sigma^{2}\right)}{\int p\left(Y|B,\sigma^{2}\right)p\left(B|\Gamma^{\prime },\sigma^{2}\right)dB}$$

Integrate $\sigma^{2}$ out, and the posterior density function of B is

(39) $$\begin{array}{ll} p\left(B|Y,\Gamma^{\prime }\right) & =\int p\left(B|Y,\Gamma^{\prime },\sigma^{2}\right)p\left(\sigma^{2}|a,b\right)d\sigma^{2}\\ & =\frac{\Gamma \left(a+LK\right){\left[1+\frac{1}{2b}\sum_{l=1}^{L}{\left(a_{l}-\mu_{l}\right)}^{H}\Sigma^{-1}\left(a_{l}-\mu_{l}\right)\right]}^{-\left(a+LK\right)}}{\Gamma \left(a\right){\left(2\pi b\right)}^{LK}{\left|\Sigma \right|}^{\frac{L}{2}}} \end{array}$$

where

(40) $$\Sigma ={\left(\Psi^{T}\Psi +\Gamma^{\prime -1}\right)}^{-1}$$

(41) $$\mu_{l}=\Sigma \Psi^{T}y_{l}$$

From (39), we can draw the conclusion that the modified formulation induces a heavy-tailed Student-t distribution on the residual noise, which improves the robustness of the algorithm.

The logarithm of the marginal likelihood function is

(42) $$\begin{array}{ll} L\left(\Gamma^{\prime }\right) & =\log p\left(Y|\Gamma^{\prime }\right)\\ & =\log\int p\left(Y|B,\sigma^{2}\right)p\left(B|\Gamma^{\prime },\sigma^{2}\right)p\left(\sigma^{2}|a,b\right)dBd\sigma^{2}\\ & =-\frac{1}{2}\sum_{l=1}^{L}\left[\left(2MN+2a\right)\log\left(y_{l}^{T}C^{-1}y_{l}+2b\right)+\log\left|C\right|\right]+\mathrm{constant} \end{array}$$

with

(43) $$C=I_{2MN}+{\Psi \Gamma }^{\prime }\Psi^{T}$$

Since the signals $y_{l},\;l=1\cdots ,L$ is group-sparse, we define

(44) $$\alpha_{i}=\gamma_{2i-1}^{\prime }=\gamma_{2i}^{\prime },i=1,2,\cdots ,K$$

and then

(45) $$\Gamma^{\prime }=\mathrm{diag}\left(\alpha \right)⊗I_{2}$$

where $\alpha =\left[\alpha_{1},\alpha_{2},\cdots ,\alpha_{K}\right]$.

### 4.3. Maximization of $L\left(\Gamma^{\prime }\right)$ to Estimate α

A most probable way to point estimate hyper-parameters α may be found via a type-II maximum likelihood procedure. Mathematically, maximize the marginal likelihood function or its logarithm (42) with respect to α. In the following substance, the FMLM algorithm is applied to maximize (42) to estimate α. Unlike the EM algorithm, the FMLM algorithm reduces the computational complexities by identifying the support space of data, i.e., the atoms in the dictionary whose corresponding values in α is non-zero.

For notational convenience, we ignore the symbol ${\;}^{\prime }$. γ and Γ in the following substance all represent $\gamma^{\prime }$ and $\Gamma^{\prime }$, respectively.

Define Ω as the set of the non-zero values in Γ and ψ as the support space of data.

(46) $$\Omega =\mathrm{diag}\left(\alpha_{\omega_{1}},\alpha_{\omega_{2}},\cdots ,\alpha_{\omega_{J}}\right)⊗I_{2}$$

(47) $$\psi =\left[\Phi_{\omega_{1}},\Phi_{\omega_{2}},\cdots ,\Phi_{\omega_{J}}\right]$$

where

(48) $$\left\{\omega_{1},\omega_{2},\cdots ,\omega_{J}\right\}=\left\{i|1\leq i\leq K,\alpha_{i}\neq 0\right\}$$

(49) Φi=Reυi−ImυiImυiReυi,i=1,2,⋯,K

and J is the number of non-zeros in α.

At the beginning of the FMLM algorithm, we initialize $\alpha^{\left(0\right)}=0$, namely, $\Omega^{\left(0\right)}=\emptyset$ and $\psi^{\left(0\right)}=\emptyset$. Then, we need to identify the support space of data in each iteration until that α converges to a steady point.

The matrix C can be decomposed into two parts.

(50) $$\begin{array}{ll} C & =I_{2MN}+\sum_{k\neq i,1\leq k\leq K}\alpha_{k}\Phi_{k}\Phi_{k}^{T}+\alpha_{i}\Phi_{i}\Phi_{i}^{T}\\ & =C_{-i}+\alpha_{i}\Phi_{i}\Phi_{i}^{T} \end{array}$$

The first term $C_{-i}=I_{2MN}+\sum_{k\neq i,1\leq k\leq K}\alpha_{k}\Phi_{k}\Phi_{k}^{T}$ contains all terms that are independent of $\alpha_{i}$, and the second term includes all the terms related to $\alpha_{i}$.

Using the Woodbury Matrix Identity, the matrix inversion and matrix determinate lemmas are expressed as

(51) $$C^{-1}=C_{-i}^{-1}-C_{-i}^{-1}\Phi_{i}{\left(\alpha_{i}^{-1}I_{2}+\Phi_{i}^{T}C_{-i}^{-1}\Phi_{i}\right)}^{-1}\Phi_{i}^{T}C_{-i}^{-1}$$

(52) $$\left|C\right|=\left|C_{-i}\right|\left|\alpha_{i}I_{2}\right|\left|\alpha_{i}^{-1}I_{2}+\Phi_{i}^{T}C_{-i}^{-1}\Phi_{i}\right|$$

Then, (42) can be expressed as

(53) $$\begin{array}{l} L\left(\Gamma \right)=-\frac{1}{2}\sum_{l=1}^{L}\left[\left(2MN+2a\right)\log\left(y_{l}^{T}C_{-i}^{-1}y_{l}+2b\right)+\log\left|C_{-i}\right|\right]\\ -\frac{1}{2}\sum_{l=1}^{L}\left[\begin{array}{cc} \; & \left(2MN+2a\right)\log⟮1,-,\frac{y_{l}^{T}C_{-i}^{-1}\Phi_{i}{\left(\alpha_{i}^{-1}I_{2}+\Phi_{i}^{T}C_{-i}^{-1}\Phi_{i}\right)}^{-1}\Phi_{i}^{T}C_{-i}^{-1}y_{l}}{y_{l}^{T}C_{-i}^{-1}y_{l}+2b}⟯\\ \; & \;+\log\left|\alpha_{i}I_{2}\right|+\log\left|\alpha_{i}^{-1}I_{2}+\Phi_{i}^{T}C_{-i}^{-1}\Phi_{i}\right| \end{array}\right]+\mathrm{constant}\\ \;=L_{-i}+L\left(\alpha_{i}\right) \end{array}$$

where $L_{-i}$ contains all terms that are independent of $\alpha_{i}$ and $L\left(\alpha_{i}\right)$ includes all the terms related to $\alpha_{i}$.

Define

(54) $${\hat{S}}_{i}≜\Phi_{i}^{T}C_{-i}^{-1}\Phi_{i},{\hat{q}}_{l,i}≜\Phi_{i}^{T}C_{-i}^{-1}y_{l},\;{\hat{g}}_{l,i}≜y_{l}^{T}C_{-i}^{-1}y_{l}+2b$$

Then, $L\left(\alpha_{i}\right)$ can be expressed as

(55) $$L\left(\alpha_{i}\right)=-\frac{1}{2}\sum_{l=1}^{L}\left[\begin{array}{l} \left(2MN+2a\right)\log⟮1-\frac{{\hat{q}}_{l,i}^{T}{\left(\alpha_{i}^{-1}I_{2}+{\hat{S}}_{i}\right)}^{-1}{\hat{q}}_{l,i}}{{\hat{g}}_{l,i}}⟯\\ +2\log\left|\alpha_{i}\right|+\log\left|\alpha_{i}^{-1}I_{2}+{\hat{S}}_{i}\right| \end{array}\right]$$

The eigenvalue decomposition (EVD) of ${\hat{S}}_{i}$

(56) $${\hat{S}}_{i}=V_{i}\Lambda_{i}V_{i}^{T}=\sum_{j=1}^{2}{\hat{s}}_{i,j}\upsilon_{i,j}\upsilon_{i,j}^{T}$$

where ${\hat{s}}_{i,j}$ and $\upsilon_{i,j}$ denote the j-th eigenvalue and eigenvector of ${\hat{S}}_{i}$ respectively. $V_{i}=\left[\upsilon_{i,1},\upsilon_{i,2}\right]$ is the eigen-matrix of ${\hat{S}}_{i}$.

Define $c_{l,i}=V_{i}^{T}{\hat{q}}_{l,i}=\left[\begin{array}{c} c_{l,i,1}\\ c_{l,i,2} \end{array}\right]$, and the formula (55) can then be expressed as

(57) $$L\left(\alpha_{i}\right)=-\frac{1}{2}\sum_{l=1}^{L}\left[\begin{array}{l} \left(2MN+2a\right)\log\left(1-\frac{\frac{\alpha_{i}c_{l,i,1}^{2}}{1+\alpha_{i}{\hat{s}}_{i,1}}+\frac{\alpha_{i}c_{l,i,2}^{2}}{1+\alpha_{i}{\hat{s}}_{i,2}}}{{\hat{g}}_{l,i}}\right)\\ +2\log\left(\alpha_{i}\right)+\sum_{j=1}^{2}\log\left(\frac{1+\alpha_{i}{\hat{s}}_{i,j}}{\alpha_{i}}\right) \end{array}\right]$$

The next step involves maximizing (57) to estimate the hyper-parameters $\alpha_{i}\;,\forall i$, and we choose only one candidate from $\alpha_{i}\;,\forall i$ that can maximize (53).

Differentiate $L\left(\alpha_{i}\right)$ with respect to $\alpha_{i}$, and set the result to zero

(58) $$\frac{\partial L\left(\alpha_{i}\right)}{\partial \alpha_{i}}=-\frac{1}{2}\sum_{l=1}^{L}\left\{\left(\frac{-\left(2MN+2a\right)⟮\frac{c_{l,i,1}^{2}}{{\left(1+\alpha_{i}{\hat{s}}_{i,1}\right)}^{2}}+\frac{c_{l,i,2}^{2}}{{\left(1+\alpha_{i}{\hat{s}}_{i,2}\right)}^{2}}⟯}{{\hat{g}}_{l,i}-\frac{\alpha_{i}c_{l,i,1}^{2}}{1+\alpha_{i}{\hat{s}}_{i,1}}-\frac{\alpha_{i}c_{l,i,2}^{2}}{1+\alpha_{i}{\hat{s}}_{i,2}}}\right)+\frac{{\hat{s}}_{i,1}}{1+\alpha_{i}{\hat{s}}_{i,1}}+\frac{{\hat{s}}_{i,2}}{1+\alpha_{i}{\hat{s}}_{i,2}}\right\}$$

which is equivalent to solve the following polynomial function

(59) $$f\left(\alpha_{i}\right)=d_{3}\alpha_{i}^{3}+d_{2}\alpha_{i}^{2}+d_{1}\alpha_{i}^{1}+d_{0}=0$$

where

(60) $$\begin{array}{cc} \; & d_{0}=\sum_{l=1}^{L}\left\{{\hat{g}}_{l,i}\left({\hat{s}}_{i,1}+{\hat{s}}_{i,2}\right)-\left(2MN+2a\right)\left(c_{l,i,1}^{2}+c_{l,i,2}^{2}\right)\right\}\\ \; & d_{1}=\sum_{l=1}^{L}\left\{\begin{array}{c} 4{\hat{g}}_{l,i}{\hat{s}}_{i,1}{\hat{s}}_{i,2}+{\hat{g}}_{l,i}\left({\hat{s}}_{i,1}^{2}+{\hat{s}}_{i,2}^{2}\right)-\left(c_{l,i,1}^{2}+c_{l,i,2}^{2}\right)\left({\hat{s}}_{i,1}+{\hat{s}}_{i,2}\right)\\ -2\left(2MN+2a\right)\left(c_{l,i,1}^{2}{\hat{s}}_{i,2}+c_{l,i,2}^{2}{\hat{s}}_{i,1}\right) \end{array}\right\}\\ & d_{2}=\sum_{l=1}^{L}\left\{\begin{array}{c} 3{\hat{g}}_{l,i}{\hat{s}}_{i,1}^{2}{\hat{s}}_{i,2}+3{\hat{g}}_{l,i}{\hat{s}}_{i,1}{\hat{s}}_{i,2}^{2}-3c_{l,i,1}^{2}{\hat{s}}_{i,1}{\hat{s}}_{i,2}-3c_{l,i,2}^{2}{\hat{s}}_{i,1}{\hat{s}}_{i,2}-c_{l,i,1}^{2}{\hat{s}}_{i,2}^{2}\\ -c_{l,i,2}^{2}{\hat{s}}_{i,1}^{2}-\left(2MN+2a+1\right)c_{l,i,1}^{2}{\hat{s}}_{i,2}^{2}-\left(2MN+2a+1\right)c_{l,i,2}^{2}{\hat{s}}_{i,1}^{2} \end{array}\right\}\\ & d_{3}=\sum_{l=1}^{L}2{\hat{g}}_{l,i}{\hat{s}}_{i,1}^{2}{\hat{s}}_{i,2}^{2}-2c_{l,i,1}^{2}{\hat{s}}_{i,1}{\hat{s}}_{i,2}^{2}-2c_{l,i,2}^{2}{\hat{s}}_{i,1}^{2}{\hat{s}}_{i,2} \end{array}$$

It has at most 3 roots found by standard root-finding algorithms. Considering that $\alpha_{i}\geq 0$ and the corresponding $\Phi_{i}$ is not in the support space when $\alpha_{i}=0$, the set of the positive roots is

(61) $$\Theta =\left\{\alpha_{i}|\alpha_{i}\in {\mathbb{R}}^{+},f\left(\alpha_{i}\right)=0\right\}$$

The optimal $\alpha_{i}^{*}$ is given by

(62) $$\alpha_{i}^{*}=\left\{\begin{array}{ccc} 0 & & if\underset{\alpha_{i}\in \Theta }{\;\max}L\left(\alpha_{i}\right)<0\\ \underset{\alpha_{i}\in \Theta }{\arg\max}L\left(\alpha_{i}\right) & & \mathrm{else} \end{array}\right.$$

If $\alpha_{i}^{*}=0$, it means the set $\left\{\alpha_{i},\Phi_{i}\right\}$ has no contribution to $L\left(\Gamma \right)$, that is to say, $L\left(\alpha_{i}^{*}\right)=0$. If $\underset{\alpha_{i}\in \Theta }{\max}L\left(\alpha_{i}\right)<0$, we need to set $\alpha_{i}^{*}=0$ because $L\left(0\right)=0>\underset{\alpha_{i}\in ℧}{\;\max}L\left(\alpha_{i}\right)$.

In the $\left(t+1\right)-\mathrm{th}$ iteration, we choose only one $\alpha_{j},1\leq j\leq K$ that can maximize (53), while fix the other $\left\{\alpha_{i}|1\leq i\leq K,i\neq j\right\}$. The sequence number j is expressed as

(63) $$j=\underset{i,1\leq i\leq K}{\arg\max}L\left(\Gamma \right)\underset{i,1\leq i\leq K}{=\arg\max}L_{-i}+L\left(\alpha_{i}^{*}\right)$$

In order to avoid calculating $L_{-i},\forall i$, we define

(64) $$\Delta L\left(i\right)≜L_{-i}+L\left(\alpha_{i}^{*}\right)-L\left(\Gamma^{\left(t\right)}\right)=L_{-i}+L\left(\alpha_{i}^{*}\right)-L_{-i}-L\left(\alpha_{i}^{\left(t\right)}\right)=L\left(\alpha_{i}^{*}\right)-L\left(\alpha_{i}^{\left(t\right)}\right)$$

Since $L\left(\Gamma^{\left(t\right)}\right)$ in the $\left(t+1\right)-\mathrm{th}$ iteration is a fixed constant, the sequence number j can also be expressed as

(65) $$j=\underset{i,1\leq i\leq K}{\mathrm{argmax}}\Delta L\left(i\right)=\underset{i,1\leq i\leq K}{\mathrm{argmax}}L\left(\alpha_{i}^{*}\right)-L\left(\alpha_{i}^{\left(t\right)}\right)$$

The next step is to change the value of $\alpha_{j}$ while fixing the other $\left\{\alpha_{i}|1\leq i\leq K,i\neq j\right\}$.

(66) $$\alpha_{j}^{\left(t+1\right)}=\alpha_{j}^{*}$$

(67) $$\alpha_{i}^{\left(t+1\right)}=\alpha_{i}^{\left(t\right)},1\leq i\leq K,i\neq j$$

If $\alpha_{j}^{\left(t+1\right)}>0$ and $\alpha_{j}^{\left(t\right)}=0$, add $\Phi_{j}$ to $\psi^{\left(t\right)}$ (i.e., $\Phi_{j}$ is not in $\psi^{\left(t\right)}$, $\psi^{\left(t+1\right)}=[\psi^{\left(t\right)},\Phi_{j}]$, $\Omega^{\left(t+1\right)}=\left[\begin{array}{cc} \Omega^{\left(t\right)} & \\ & \alpha_{j}^{\left(t+1\right)}⊗I_{2} \end{array}\right]$); if $\alpha_{j}^{\left(t+1\right)}>0$ and $\alpha_{j}^{\left(t\right)}>0$ (i.e., $\Phi_{j}$ is already in $\psi^{\left(t\right)}$, $\psi^{\left(t+1\right)}=\psi^{\left(t\right)}$), replace $\alpha_{j}^{\left(t\right)}$ with $\alpha_{j}^{\left(t+1\right)}$ in $\Omega^{\left(t\right)}$; if $\alpha_{j}^{\left(t+1\right)}=0$ and $\alpha_{j}^{\left(t\right)}>0$, delete $\Phi_{j}$ from $\psi^{\left(t\right)}$ and delete $\alpha_{j}^{\left(t\right)}$ from $\Omega^{\left(t\right)}$; and if $\alpha_{j}^{\left(t+1\right)}=0$ and $\alpha_{j}^{\left(t\right)}=0$ (i.e., $\Gamma^{\left(t+1\right)}=\Gamma^{\left(t\right)}$), stop iteration because the procedure converges to steady state. Finally, $\psi^{*}$ is the exact support space of the data and $\Omega^{*}$ is the set of the non-zero values in the exact Γ where the symbol * represents the stopping criterion.

### 4.4. Fast Computation of $\left\{{\hat{S}}_{i},{\hat{q}}_{l,i},{\hat{g}}_{l,i}\right\}$

In each iteration, we need to calculate the matrix inversion of all $C_{-i},\;\forall i$ when updating $\left\{{\hat{S}}_{i},{\hat{q}}_{l,i},{\hat{g}}_{l,i}\right\}$. In order to reduce computation complexities, we need a fast means to update $\left\{{\hat{S}}_{i},{\hat{q}}_{l,i},{\hat{g}}_{l,i}\right\}$.

Define

(68) $$S_{i}≜\Phi_{i}^{T}C^{-1}\Phi_{i},\;q_{l,i}≜\Phi_{i}^{T}C^{-1}y_{l},\;g_{l}≜y_{l}^{T}C^{-1}y_{l}+2b$$

If we can calculate $\left\{{\hat{S}}_{i},{\hat{q}}_{l,i},{\hat{g}}_{l,i}\right\}$ with $\left\{S_{i},q_{l,i},g_{l}\right\}$, the computational complexities can be reduced a lot because we only calculate the matrix inversion of C.

Substituting (51) into (68), we can arrive at the following formula:

(69) $$\begin{array}{ll} S_{i} & =\Phi_{i}^{T}C^{-1}\Phi_{i}\\ & =\Phi_{i}^{T}\left(C_{-i}^{-1}-C_{-i}^{-1}\Phi_{i}{\left(\alpha_{i}^{-1}I_{2}+\Phi_{i}^{T}C_{-i}^{-1}\Phi_{i}\right)}^{-1}\Phi_{i}^{T}C_{-i}^{-1}\right)\Phi_{i}\\ & ={\hat{S}}_{i}-{\hat{S}}_{i}{\left(\alpha_{i}^{-1}I_{2}+{\hat{S}}_{i}\right)}^{-1}{\hat{S}}_{i}\\ & =V_{i}\left[\begin{array}{cc} \frac{{\hat{S}}_{i,1}}{1+\alpha_{i}{\hat{S}}_{i,1}} & \\ & \frac{{\hat{S}}_{i,2}}{1+\alpha_{i}{\hat{S}}_{i,2}} \end{array}\right]V_{i}^{T} \end{array}$$

We can draw the conclusion that the eigen-matrix of $S_{i}$ is the same as that of ${\hat{S}}_{i}$ from (56) and (69). The EVD of $S_{i}$ can be also expressed as

(70) $$S_{i}=V_{i}\left[\begin{array}{cc} S_{i,1} & \\ & S_{i,2} \end{array}\right]V_{i}^{T}$$

where $S_{i,j}$ denotes the j-th eigenvalue of $S_{i}$.

(71) $$S_{i,j}=\frac{{\hat{S}}_{i,j}}{1+\alpha_{i}{\hat{S}}_{i,j}},j=1,2$$

Thus, ${\hat{S}}_{i}$ can be obtained via the EVD of $S_{i}$

(72) $${\hat{S}}_{i}=V_{i}\left[\begin{array}{cc} \frac{S_{i,1}}{1-\alpha_{i}S_{i,1}} & \\ & \frac{S_{i,2}}{1-\alpha_{i}S_{i,2}} \end{array}\right]V_{i}^{T}$$

${\hat{q}}_{l,i}$ and ${\hat{g}}_{l,i}$ can be also computed with $q_{l,i}$ and $g_{l}$, respectively, via the EVD of $S_{i}$.

(73) $$\begin{array}{ll} q_{l,i} & =\Phi_{i}^{T}C^{-1}y_{l}\\ & =\Phi_{i}^{T}\left(C_{-i}^{-1}-C_{-i}^{-1}\Phi_{i}{\left(\alpha_{i}^{-1}I_{2}+\Phi_{i}^{T}C_{-i}^{-1}\Phi_{i}\right)}^{-1}\Phi_{i}^{T}C_{-i}^{-1}\right)y_{l}\\ & ={\hat{q}}_{l,i}-{\hat{S}}_{i}{\left(\alpha_{i}^{-1}I_{2}+{\hat{S}}_{i}\right)}^{-1}{\hat{q}}_{l,i}\\ & =\left(I_{2}-{\hat{S}}_{i}{\left(\alpha_{i}^{-1}I_{2}+{\hat{S}}_{i}\right)}^{-1}\right){\hat{q}}_{l,i} \end{array}$$

Thus,

(74) $${\hat{q}}_{l,i}={\left(I_{2}-{\hat{S}}_{i}{\left(\alpha_{i}^{-1}I_{2}+{\hat{S}}_{i}\right)}^{-1}\right)}^{-1}q_{l,i}=V_{i}\left[\begin{array}{cc} \frac{1}{1-\alpha_{i}s_{i,1}} & \\ & \frac{1}{1-\alpha_{i}s_{i,2}} \end{array}\right]V_{i}^{T}q_{l,i}$$

Similarly,

(75) $$\begin{array}{ll} g_{l} & =y_{i}^{T}\left(C_{-i}^{-1}-C_{-i}^{-1}\Phi_{i}{\left(\alpha_{i}^{-1}I_{2}+\Phi_{i}^{T}C_{-i}^{-1}\Phi_{i}\right)}^{-1}\Phi_{i}^{T}C_{-i}^{-1}\right)y_{l}+2b\\ & ={\hat{g}}_{l,i}-{\hat{q}}_{l,i}^{T}{\left(\alpha_{i}^{-1}I_{2}+{\hat{S}}_{i}\right)}^{-1}{\hat{q}}_{l,i}\\ & ={\hat{g}}_{l,i}-{\hat{q}}_{l,i}^{T}V_{i}\left[\begin{array}{cc} \alpha_{i}\left(1-\alpha_{i}S_{i,1}\right) & \\ & \alpha_{i}\left(1-\alpha_{i}S_{i,2}\right) \end{array}\right]V_{i}^{T}{\hat{q}}_{l,i} \end{array}$$

Using (74), we can obtain

(76) $${\hat{g}}_{l,i}=g_{l}+q_{l,i}^{T}V_{i}\left[\begin{array}{cc} \frac{\alpha_{i}}{1-\alpha_{i}s_{i,1}} & \\ & \frac{\alpha_{i}}{1-\alpha_{i}s_{i,2}} \end{array}\right]V_{i}^{T}q_{l,i}$$

Therefore, $\left\{{\hat{S}}_{i},{\hat{q}}_{l,i},{\hat{g}}_{l,i}\right\}$ can be obtained from $\left\{S_{i},q_{l,i},g_{l}\right\}$ using (72), (74) and (76).

Compared with (68), there is another approach that requires fewer computational complexities to calculate $\left\{S_{i},q_{l,i},g_{l}\right\}$. The formula (43) is equivalent to

(77) $$C=I_{2MN}+\psi \Omega \psi^{T}$$

With matrix inversion lemmas and (77), it is fast and more convenient to update $\left\{S_{i},q_{l,i},g_{l}\right\}$ with the following formulae compared with (68).

(78) $$\begin{array}{l} S_{i}=\Phi_{i}^{T}\Phi_{i}-\Phi_{i}^{T}\psi \Sigma \psi^{T}\Phi_{i}\\ q_{l,i}=\Phi_{i}^{T}y_{l}-\Phi_{i}^{T}\psi \Sigma \psi^{T}y_{l}\\ g_{l}=y_{l}^{T}y_{l}-y_{l}^{T}\psi \Sigma \psi^{T}y_{l}+2b \end{array}$$

where Σ herein represents the covariance of the non-zeros in Γ.

(79) $$\Sigma ={\left(\psi^{T}\psi +{\left\{\Omega \right\}}^{-1}\right)}^{-1}$$

Additionally, the mean of the non-zeros in Γ herein is expressed as

(80) $$\mu_{l}=\Sigma \psi^{T}y_{l},l=1,\cdots ,L$$

The computational complexities are measured in terms of the number of multiplications. Assuming that the dimension of ψ is 2MN×r, r is not a fixed value in each iteration but satisfies the condition r≪MN<K because the measurements are sparse. When we calculate $S_{i}$ with (68), the computation complexities are in order of $o\left({\left(2MN\right)}^{3}+8{\left(MN\right)}^{2}+8MN\right)$. With (78), the computation complexities of calculating $S_{i}$ are in order of $o\left(r^{3}+16MN+8MNr+2r^{2}\right)$. It is apparent that the latter operation is faster and more convenient.

Since the measurements are sparse, the dimension of the matrix $\psi^{T}\psi +{\left\{\Omega \right\}}^{-1}$ in (79) is far smaller than the dimension of $\Psi^{T}\Psi +\Gamma^{\prime -1}$ in (41). The proposed M-FMLM-STAP algorithm identifies the support space of data to reduce the effective problem dimensions drastically due to sparsity. Therefore, the proposed algorithm has tremendous potential for real- time operation.

The proposed M-FMLM-STAP algorithm is shown in Algorithm 2. The detailed update formulae are shown in the Appendix A.
**Algorithm 2:** Pseudocode for M-FMLM-STAP algorithm.Step 1: Input: the original data X, the original dictionary D and a=b=0.Step 2: y=ReX;ImX and Ψ.Step 3: Initialize:$C=C_{-i}=I$ α=0 and ψ=∅.Step 4: while not converged do     Choose only one candidate $j\in \left\{1,2,\cdots \;,K\right\}$ and find optimal $\alpha_{j}$ using (65).     If $\alpha_{j}^{\left(t+1\right)}>0$ and $\alpha_{j}^{\left(t\right)}=0$, then        $\psi^{\left(t+1\right)}=[\psi^{\left(t\right)},\Phi_{j}]$, and $\Omega^{\left(t+1\right)}=\left[\begin{array}{cc} \Omega^{\left(t\right)} & \\ & \alpha_{j}^{\left(t+1\right)}⊗I_{2} \end{array}\right]$.      Otherwise, if $\alpha_{j}^{\left(t+1\right)}>0$ and $\alpha_{j}^{\left(t\right)}>0$, then        $\psi^{\left(t+1\right)}=\psi^{\left(t\right)}$, and replace $\alpha_{j}^{\left(t\right)}$ with $\alpha_{j}^{\left(t+1\right)}$.      Otherwise, if $\alpha_{j}^{\left(t+1\right)}>0$ and $\alpha_{j}^{\left(t\right)}>0$, then        delete $\Phi_{j}$ from $\psi^{\left(t\right)}$, and delete $\alpha_{j}^{\left(t\right)}$ from $\Omega^{\left(t\right)}$.      end      Update Σ,μ,$S_{i}$,$q_{l,i}$ and $g_{l}$ referring to Appendix A.    end whileStep 5: Estimate the CNCM R by     $R=\frac{1}{L}\sum_{l=1}^{L}\sum_{i=1}^{J}\left({\left\|\mu_{l,2i-1}^{*}\right\|}^{2}+{\left\|\mu_{l,2i}^{*}\right\|}^{2}\right)\upsilon_{\omega_{i}}\upsilon_{\omega_{i}}^{H}+\beta Ι$
   where the vector $\upsilon_{\omega_{i}}$ is the $\omega_{i}-\mathrm{th}$ column of D, J is the number of non-zeros in $\alpha^{*}$ and β is a load factor. The symbol * represents the stopping criterion.Step 6: Compute the space-time adaptive weight w using (7).Step 7: The output of M-FMLM-STAP is $Z=w^{H}X$.

The main symbols aforementioned are listed in Table 1.

##  5. Complexity Analysis and Convergence Analysis

### 5.1. Complexity Analysis

Here, we compare the computational complexities of the proposed M-FMLM-STAP algorithm and the M-SBL-STAP algorithm for a single iteration. The computational complexities are measured in terms of the number of multiplications.

First, we analyze the computational complexities of the M-SBL-STAP algorithm. It is apparent that the main computational complexities of the M-SBL-STAP algorithm are related to the formulae (17) and (18). Noting that Λ in (17) is a diagonal matrix, the computational complexities of (17) are in the order of $o\left(K^{3}+K^{2}MN\right)$. The computational complexities of (18) are in the order of $o\left(K^{2}MN+KMNL\right)$. Thus, the computational complexities of the M-SBL-STAP algorithm are in the order of $o\left(K^{3}+2K^{2}MN+KMNL\right)$.

Second, we analyze the computational complexities of the M-FMLM-STAP algorithm. Noting that the dimension of $S_{i}$ is 2×2, the computational complexities of the EVD of $S_{i}$ are small enough to be ignored, that is to say, the computational complexities to calculate $\left\{{\hat{S}}_{i},{\hat{q}}_{l,i},{\hat{g}}_{l,i}\right\}$ with $\left\{S_{i},q_{l,i},g_{l}\right\}$ can be ignored. Meanwhile, the computational complexities to solve the polynomial function (59), ∀i,1≤i≤K and to find the sequence number (65) are in direct proportion to K, which are also small enough to be ignored. Thus, the main computational complexities of the M-FMLM-STAP algorithm are used to update $\left\{S_{i},q_{l,i},g_{l},\Sigma ,\mu \right\}$. Assuming that the dimension of ψ is 2MN×r, r is not a fixed value in all iterations. However, the condition r≪MN<K is satisfied because the measurements are sparse. The computational complexities of the term $\Phi_{i}^{T}-\Phi_{i}^{T}\psi \Sigma \psi^{T}$ are in the order of $o\left(8MNr+2r^{2}\right)$. Thus, ∀i and ∀l, the computational complexities of (78), are in the order of $o\left(\left(8MNr+2r^{2}\right)K+8MNK+4MNLK+\left(4MNr+r^{2}+4MN\right)L\right)$. The computational complexities of (79) and (80) are in the order of $o\left(2MNr^{2}+r+r^{3}\right)$ and $o\left(2MNr^{2}+2MNrL\right)$, respectively. To sum up, ignore the low-order computational complexities, and the computational complexities of the M-FMLM-STAP algorithm for a single iteration are in the order of $o\left(8MNrK+4MNLK+6MNrL\right)$.

**Figure 1:**
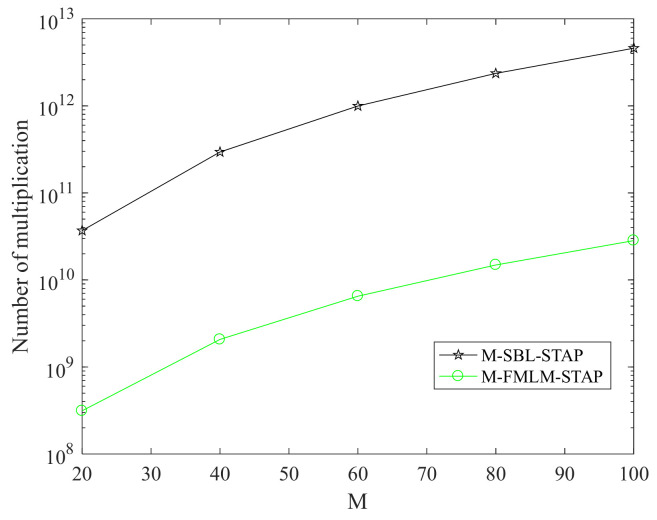
Computational complexity versus the number of pluses for a single iteration.

Figure 1 illustrates the complexity requirements of two algorithms for a single iteration. It shows the computational complexities to the number of pluses M for a single iteration. Suppose that $\varphi_{S}=\varphi_{d}=4$, N=10 and L=6. Although the value r is unknown and unfixed in each iteration, we can suppose that it is twice as much to the rank of clutter in the current iteration. We can draw the conclusion that the computational complexities of the M-SBL-STAP algorithm are far more than that of the M-FMLM-STAP algorithm for a single iteration.

### 5.2. Convergence Analysis

It is obvious that the logarithm of the marginal likelihood function $L\left(\Gamma \right)$ has upper bound [31]. From (62), in the t-th iteration, $L\left(\alpha_{i}^{*}\right)$ is the maximal value of $L\left(\alpha_{i}\right)$, that is to say, $L\left(\alpha_{i}^{*}\right)\geq L\left(\alpha_{i}^{\left(t\right)}\right)$. Therefore, the condition $L\left(\Gamma^{\left(t+1\right)}\right)\geq L\left(\Gamma^{\left(t\right)}\right),\forall t$ is promised in each iteration referring to (65). Thus, the convergence performance of our proposed algorithm is guaranteed.

## 6. Performance Assessment

In this section, we firstly verify the performance of the loading sample matrix inversion (LSMI) algorithm, the multiple orthogonal matching pursuit (M-OMP-STAP) algorithm [32], the M-SBL-STAP algorithm and the proposed M-FMLM-STAP algorithm with the simulated data of side-looking ULA in the ideal case and the array errors case. The first two algorithms are common approaches in STAP. We then assess the performance of the M-SBL-STAP algorithm and the proposed M-FMLM-STAP algorithm with the Mountain-Top data. We utilize the improvement factor (IF) and signal to interference plus noise ratio (SINR) loss as two measurements of performance, which are expressed as

(81) $$\mathrm{IF}=\frac{{\left|w^{H}s\right|}^{2}}{w^{H}Rw}\frac{\mathrm{tr}\left(R\right)}{s^{H}s}$$

(82) $$L_{SINR}=\frac{\sigma^{2}}{MN}\frac{{\left|w^{H}s\right|}^{2}}{w^{H}Rw}$$

where R is the exact CNCM of the CUT.

### 6.1. Simulated Data

The parameters of a side-looking phased array radar are listed in Table 2. The 600th range gate is chosen to be CUT. According to the parameters of the radar system, the slope of the clutter ridge is 1. We set the resolution scales $\varphi_{S}=5$ and $\varphi_{d}=5$. Total of six training samples are utilized for two algorithms and the parameters a=b=0.

**Figure 2:**
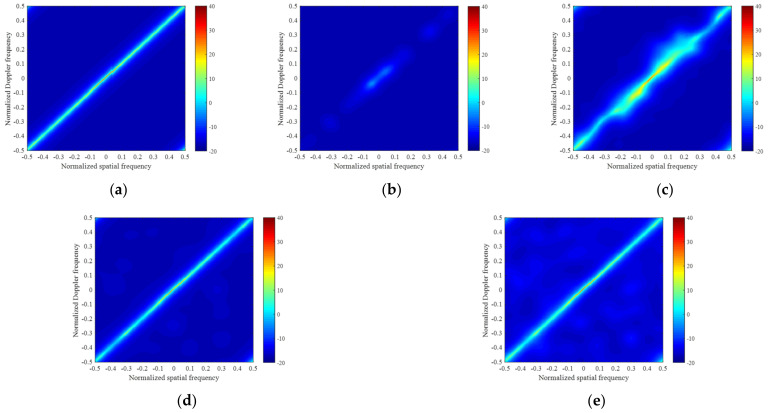
Angle-Doppler clutter power spectrums in the ideal case: (**a**) exact spectrum calculated by the ideal CNCM; (**b**) sparse spectrum estimated by LSMI; (**c**) sparse spectrum estimated by M-OMP-STAP; (**d**) sparse spectrum estimated by M-SBL-STAP; and (**e**) sparse spectrum estimated by M-FMLM-STAP.

**Figure 3:**
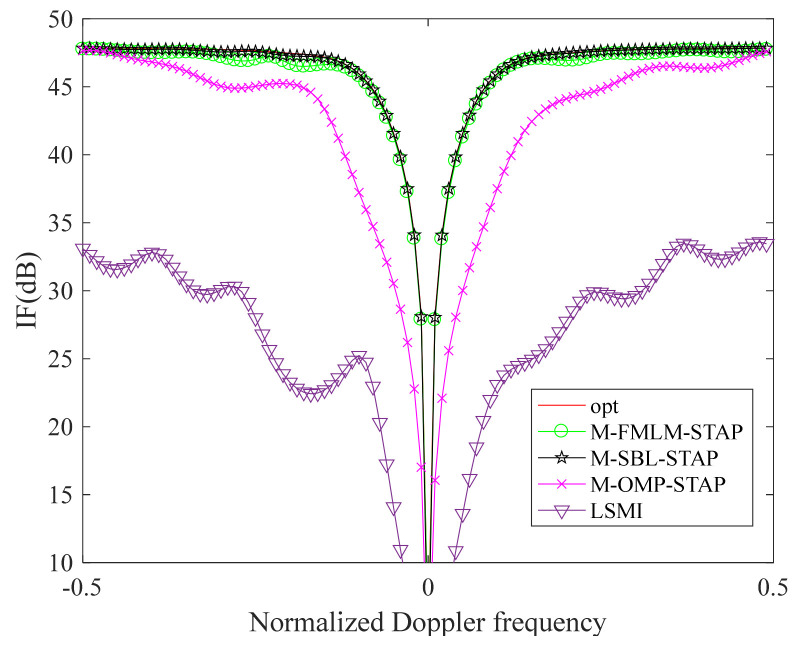
IF curves in the ideal case.

In Figure 2, there are five clutter power spectrum figures in the framework of the side-looking ULA in the ideal case. Figure 2a–e show the clutter power spectrums estimated by the exact CNCM, the LSMI algorithm, the M-OMP-STAP algorithm, the M-SBL-STAP algorithm and the M-FMLM-STAP algorithm, respectively. We note that the clutter spectrums obtained by the M-SBL-STAP algorithm and the M-FMLM-STAP algorithms are closer to the exact spectrum both on the location and power than the other algorithms in the ideal case. As shown in Figure 3, the IF factor curve achieved by the M-SBL-STAP algorithm is a litter better than that achieved by the M-FMLM-STAP algorithm because the latter algorithm is sensitive to noise fluctuation. However, the latter algorithm needs much fewer computational complexities, and the loss on performance can be offset by the decrease on the computational complexities. Using a computer equipped with two Inter(R) Xeon(R) Gold 6140 CPU @2.30 GHz 2.29 GHz processors, the former spends more than 40 s and the latter spends less than 4 s at average. The larger the value of K, the bigger gap of computational complexities between the M-SBL-STAP algorithm and the M-FMLM-STAP algorithm.

**Figure 4:**
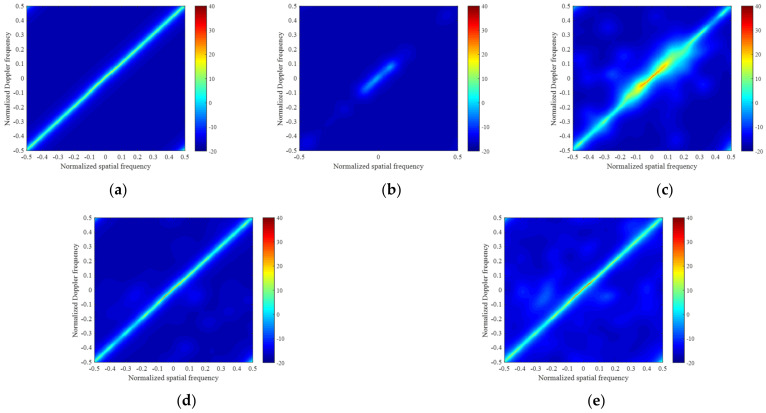
Angle-Doppler clutter power spectrums in the non-ideal case with array errors: (**a**) exact spectrum calculated by the exact CNCM; (**b**) sparse spectrum estimated by LSMI; (**c**) sparse spectrum estimated by M-OMP-STAP; (**d**) sparse spectrum estimated by M-SBL-STAP; and (**e**) sparse spectrum estimated by M-FMLM-STAP.

**Figure 5:**
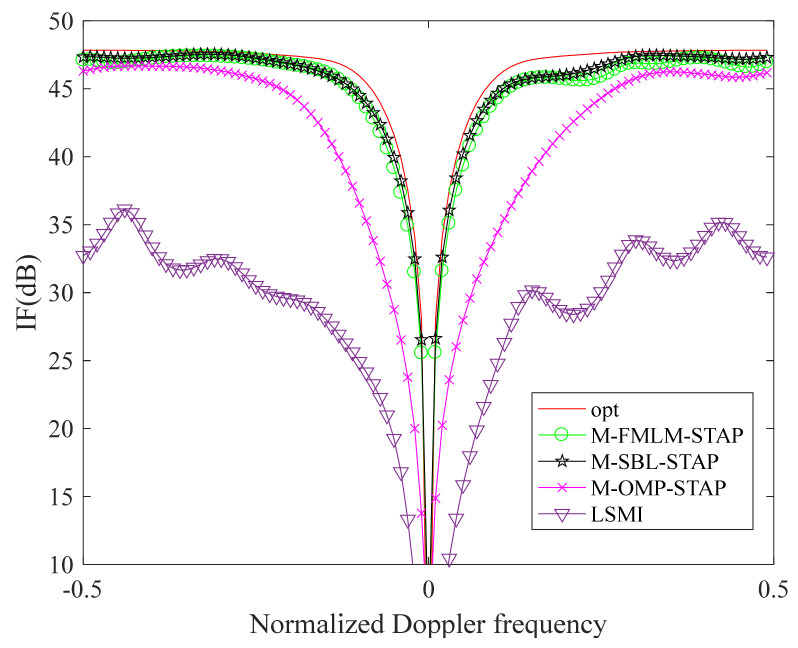
IF curves in the non-ideal case.

In Figure 4, we apply four approaches in the non-ideal case with amplitude Gaussian error (standard deviation 0.03) and phase random error (standard deviation $2^{\circ }$). The error is the same at all direction. Figure 4a–e show the clutter power spectrums estimated by the exact CNCM, the LSMI algorithm, the M-OMP-STAP algorithm, the M-SBL-STAP algorithm and the M-FMLM-STAP algorithm, respectively. We note that the clutter spectrums obtained by the M-SBL-STAP algorithm and the M-FMLM-STAP algorithm are much closer to the exact spectrum, both in terms of the location and power in the non-ideal case. As shown in Figure 5, the notch of the IF factor curve achieved by the M-SBL-STAP algorithm is close to that achieved by the M-FMLM-STAP algorithm, which means the performance of two algorithms is about the same in the non-ideal case. However, the running time of the latter is much less than that of the former.

**Figure 6:**
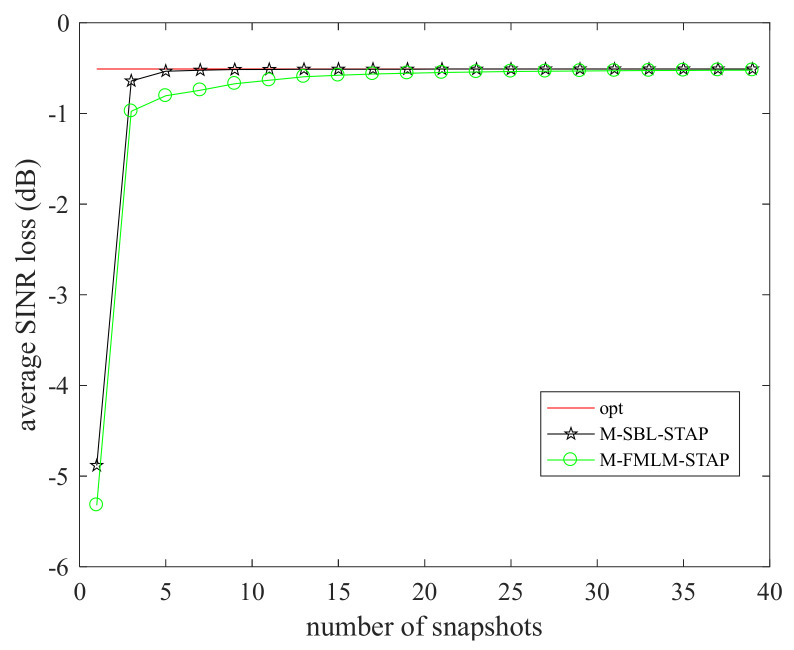
Average SINR loss to the number of training samples.

In Figure 6, the average SINR loss, defined as the mean of the SINR loss values in the entire normalized Doppler frequency range to the number of training samples of the M-SBL-STAP algorithm and the M-FMLM-STAP algorithm, is presented. The curve of the average SINR loss achieved by the M-SBL-STAP algorithm is a litter better than that achieved by the M-FMLM-STAP algorithm within 0.5 dB, which can be ignored in practice. However, the gap on computational complexities of two algorithms will be larger as the DOF of the radar system increases. All presented results are averaged over 100 independent Monte Carlo runs.

### 6.2. Measured Data

**Figure 7:**
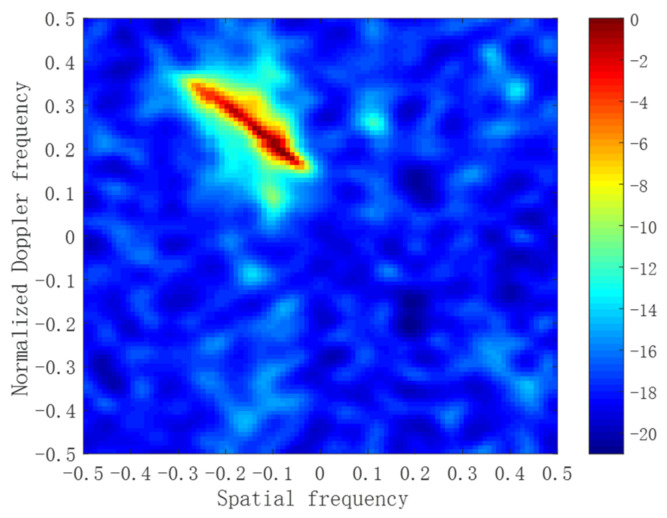
Estimated clutter Capon spectrum with all samples.

We apply the M-SBL-STAP algorithm and the M-FMLM-STAP algorithm to the public available Mountain-Top set, i.e., t38pre01v1 CPI6 [33]. In this data file, the number of array elements and coherent pulses are 14 and 16, respectively. There are 403 range cells, and the clutter locates around 245° relative to the true North. The target is located in 147th range cell, and the azimuth angle is 275° to the true North. The normalized target Doppler frequency is 0.25. The estimated clutter Capon spectrum utilizing all 403 range cells is provided in Figure 7.

**Figure 8:**
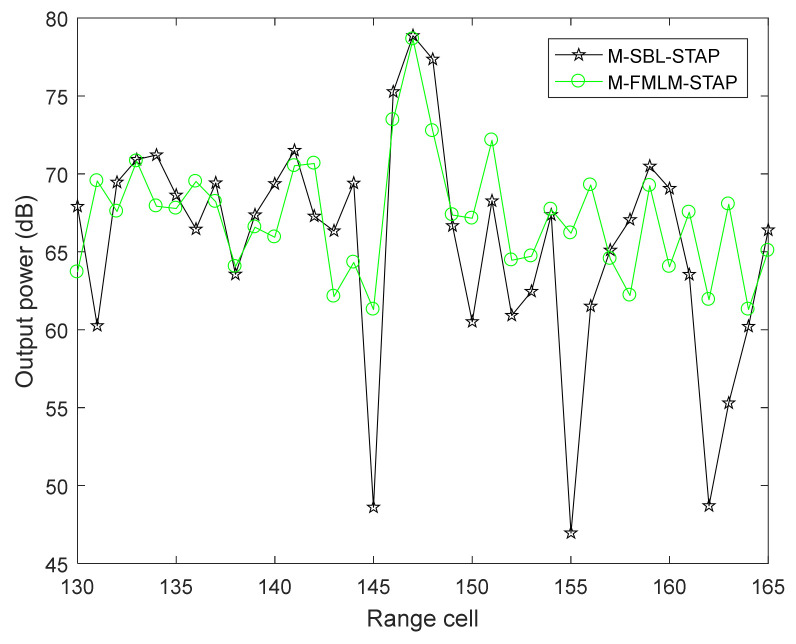
STAP output power to the range cell of two algorithms.

Figure 8 depicts the STAP output power of the M-SBL-STAP algorithm and the M-FMLM-STAP algorithm in the range cells from 130 to 165, and 10 range cells out of 20 range cells located next to the CUT are selected as training samples. The target locates at 147th range cell and can be detected by two algorithms. Obviously, the detection performance of the proposed M-FMLM-STAP algorithm is close to that of M-SBL-STAP algorithm. However, the operation time of the former algorithm is much less than that of the latter algorithm. Therefore, the proposed algorithm is applicable in practice.

## 7. Conclusions

In this paper, we have extended the real-valued multitask compressive sensing technique to suppress complex-valued heterogeneous clutter for airborne radar. Unlike the conventional M-SBL-STAP algorithm, the proposed algorithm avoids the inversion of a K×K matrix at each iteration to guarantee real-time operations. We integrate the noise $\sigma^{2}$ out instead of estimating $\sigma^{2}$ to overcome the problem that we have no access to obtain the accurate value of $\sigma^{2}$. The complex-valued multitask clutter data are translated into group real-valued sparse signals in the article to suppress clutter because the modified hierarchical model is not suitable to complex-valued signals. At the end, simulation results demonstrate the high computational efficiency and the great performance of the proposed algorithm.

## Figures and Tables

**Table 1:** The main symbols.

Symbols	Parameters	Symbols	Parameters
X	The original data	Y	The new data
D	The original dictionary	Ψ	The new dictionary
A	The original coefficient matrix	B	The new coefficient matrix
Γ	The original hyper-parameter	$\Gamma^{\prime }$	The new hyper-parameter
Σ	The covariance of coefficient	μ	The mean of coefficient
Ω	The set of the non-zero values in $\Gamma^{\prime }$	ψ	The support space of data
$\left\{{\hat{S}}_{i},{\hat{q}}_{l,i},{\hat{g}}_{l,i}\right\}$	See (54)	$\left\{S_{i},q_{l,i},g_{l}\right\}$	See (68)
$\Phi_{i}$	See (49)		

**Table 2:** Radar system parameters.

Symbols	Parameters	Value
λ	Wavelength	0.3 m
d	Distance between elements	0.15 m
ϑ	Platform velocity	150 m/s
H	Platform height	9000 m
M	Number of pulses	8
N	Number of channels	8
$f_{\mathrm{PRF}}$	Pulse repetition frequency	2000 Hz
$f_{S}$	Range sampling frequency	2.5 MHz
θ	Perspective angle	90°
CNR	Clutter to noise ratio	30 dB

## Appendix A

For notational convenience, we ignore the symbol $\left(t\right)$ in the t-th iteration and use ~ to represent the $\left(t+1\right)-\mathrm{th}$ iteration.

### Appendix A.1. Adding a New Basis Group Function (${\tilde{\alpha }}_{i}>0\;\&\&\;\alpha_{i}=0$)

When $\alpha_{i}=0$, $C=C_{-i}$.

(A1) $$\Delta L\left(i\right)=-\frac{1}{2}\sum_{l=1}^{L}\left\{\begin{array}{l} \left(2NM+2a\right)\log⟮1-\frac{q_{l,i}^{T}{\left({\tilde{\alpha }}_{i}^{-1}I_{2}+S_{i}\right)}^{-1}q_{l,i}}{g_{l}}⟯\\ +\log\left|{\tilde{\alpha }}_{i}I_{2}\right|+\log\left|{\tilde{\alpha }}_{i}^{-1}I_{2}+S_{i}\right| \end{array}\right\}$$

(A2) $$\tilde{\Sigma }=\left[\begin{array}{cc} \Sigma +\Sigma \psi^{T}\Phi_{i}Z\Phi_{i}^{T}\psi \Sigma & -\Sigma \psi^{T}\Phi_{i}Z\\ -{\left(\Sigma \psi^{T}\Phi_{i}Z\right)}^{T} & Z \end{array}\right]$$

(A3) $${\tilde{\mu }}_{l}=\left[\begin{array}{c} \mu_{l}-\Sigma \psi^{T}\Phi_{i}Zq_{l,i}\\ Zq_{l,i} \end{array}\right]$$

(A4) $${\tilde{S}}_{i}=S_{i}-S_{i}ZS_{i}=S_{i}-\Phi_{i}^{T}E_{i}ZE_{i}^{T}\Phi_{i}$$

(A5) $${\tilde{q}}_{l,i}=q_{l,i}-S_{i}Zq_{l,i}=q_{l,i}-\Phi_{i}^{T}E_{i}Zq_{l,i}$$

(A6) $${\tilde{g}}_{l}=g_{l}-q_{l,i}^{T}Zq_{l,i}=g_{l}-y_{l}^{T}E_{i}ZE_{i}^{T}y_{l}$$

where $Z={\left({\tilde{\alpha }}_{i}^{-1}I_{2}+S_{i}\right)}^{-1}$ and $E_{i}=\Phi_{i}-\psi \Sigma \psi^{T}\Phi_{i}$.

### Appendix A.2. Re-Estimating a Basis Group Function (${\tilde{\alpha }}_{i}>0\;\&\&\;\alpha_{i}>0$)

i represents the index of the i-th group in the whole dictionary matrix, and g represents the position index of the above-mentioned i-th group in the used basis matrix ψ. ${\tilde{\alpha }}_{i}$ is the hyper-parameter re-estimated, $\Delta \alpha_{i}={\tilde{\alpha }}_{i}-\alpha_{i}$, $\Sigma_{g}=\Sigma \left(:,2g-1:2g\right)$, and $\Sigma_{gg}=\Sigma \left(2g-1:2g,2g-1:2g\right)$.

(A7) $$\Delta L\left(i\right)=-\frac{1}{2}\sum_{l=1}^{L}\left\{\begin{array}{l} \left(2NM+2a\right)\log⟮1-\frac{{\hat{q}}_{l,i}^{T}{\left({\tilde{\alpha }}_{i}^{-1}I_{2}+{\hat{S}}_{i}\right)}^{-1}{\hat{q}}_{l,i}}{{\hat{g}}_{l,i}}⟯\\ +\log\left|{\tilde{\alpha }}_{i}I_{2}\right|+\log\left|{\tilde{\alpha }}_{i}^{-1}I_{2}+{\hat{S}}_{i}\right|\\ -\left(2NM+2a\right)\log⟮1-\frac{{\hat{q}}_{l,i}^{T}{\left(\alpha_{i}^{-1}I_{2}+{\hat{S}}_{i}\right)}^{-1}{\hat{q}}_{l,i}}{{\hat{g}}_{l,i}}⟯\\ -\log\left|\alpha_{i}I_{2}\right|-\log\left|\alpha_{i}^{-1}I_{2}+{\hat{S}}_{i}\right| \end{array}\right\}$$

(A8) $$\tilde{\Sigma }=\Sigma -\Sigma_{g}G\Sigma_{g}^{T}$$

(A9) $$\tilde{\mu }=\mu -\Sigma_{g}G\mu_{g}$$

(A10) $${\tilde{S}}_{i}=S_{i}+\Phi_{i}^{T}\psi \Sigma_{g}G\Sigma_{g}^{T}\psi^{T}\Phi_{i}$$

(A11) $${\tilde{q}}_{l,i}=q_{l,i}+\Phi_{i}^{T}\psi \Sigma_{g}G\Sigma_{g}^{T}\psi^{T}y_{l}$$

(A12) $${\tilde{g}}_{l}=g_{l}+y_{l}^{T}\psi \Sigma_{g}G\Sigma_{g}^{T}\psi^{T}y_{l}$$

where $G={⟮-\frac{\alpha_{i}{\tilde{\alpha }}_{i}}{\Delta \alpha_{i}}I_{2}+\Sigma_{gg}⟯}^{-1}$ and $\mu_{g}=\Sigma_{g}^{T}\psi^{T}y$ denotes the g-th 2×1 sub-vector of μ.

### Appendix A.3. Deleting a Basis Group Function (${\tilde{\alpha }}_{i}=0\;\&\&\;\alpha_{i}>0$)

i represents the index of the i-th group in the whole dictionary matrix, and g represents the position index of the above-mentioned i-th group, which is removed in the used basis matrix ψ.

(A13) $$\Delta L\left(i\right)=\frac{1}{2}\sum_{l=1}^{L}\left[\begin{array}{l} \left(2NM+2a\right)\log⟮1-\frac{{\hat{q}}_{l,i}^{T}{\left(\alpha_{i}^{-1}I_{2}+{\hat{S}}_{i}\right)}^{-1}{\hat{q}}_{l,i}}{{\hat{g}}_{l,i}}⟯\\ +\log\left|\alpha_{i}I_{2}\right|+\log\left|\alpha_{i}^{-1}I_{2}+{\hat{S}}_{i}\right| \end{array}\right]$$

(A14) $$\tilde{\Sigma }=\Sigma -\Sigma_{g}\Sigma_{gg}^{-1}\Sigma_{g}^{T}$$

(A15) $$\tilde{\mu }=\mu -\Sigma_{g}\Sigma_{gg}^{-1}\mu_{g}$$

(A16) $${\tilde{S}}_{i}=S_{i}+\Phi_{i}^{T}\psi \Sigma_{g}\Sigma_{gg}^{-1}\Sigma_{g}^{T}\psi^{T}\Phi_{i}$$

(A17) $${\tilde{q}}_{l,i}=q_{l,i}+\Phi_{i}^{T}\psi \Sigma_{g}\Sigma_{gg}^{-1}\Sigma_{g}^{T}\psi^{T}y_{l}$$

(A18) $${\tilde{g}}_{l}=g_{l}+y_{l}^{T}\psi \Sigma_{g}\Sigma_{gg}^{-1}\Sigma_{g}^{T}\psi^{T}y_{l}$$
